# Singular Case of Hydrophobia

**Published:** 1853-07

**Authors:** 


*Singular Case of Hydrophobia.*—Last Christmas Anne Turner, aged seven, and residing at Birkenhead, was bitten by a dog, which was subsequently killed. No bad symptoms betrayed themselves until lately, when upon her mother coughing, the child screamed and became ill. Medical gentlemen were instantly called in, who, suspecting the character of the disease, blew upon the body at the distance of six feet which caused her to renew her screams; yet she had no delirium, nor objections to liquids, and her memory was good. A few days before her death her motions were dark and fœtid, her manner foolish, and sometimes she became rather furious, threatening to injure and bite her parents. At length death released her from her sufferings.—*ibid.*

